# Extraction of Coronary Atherosclerotic Plaques From Computed Tomography Imaging: A Review of Recent Methods

**DOI:** 10.3389/fcvm.2021.597568

**Published:** 2021-02-10

**Authors:** Haipeng Liu, Aleksandra Wingert, Jian'an Wang, Jucheng Zhang, Xinhong Wang, Jianzhong Sun, Fei Chen, Syed Ghufran Khalid, Jun Jiang, Dingchang Zheng

**Affiliations:** ^1^Research Centre for Intelligent Healthcare, Coventry University, Coventry, United Kingdom; ^2^Faculty of Health, Education, Medicine, and Social Care, Anglia Ruskin University, Chelmsford, United Kingdom; ^3^Department of Cardiology, School of Medicine, The Second Affiliated Hospital, Zhejiang University, Hangzhou, China; ^4^Department of Clinical Engineering, School of Medicine, The Second Affiliated Hospital, Zhejiang University, Hangzhou, China; ^5^Department of Radiology, School of Medicine, The Second Affiliated Hospital, Zhejiang University, Hangzhou, China; ^6^Department of Electrical and Electronic Engineering, Southern University of Science and Technology, Shenzhen, China

**Keywords:** coronary artery disease, atherosclerosis, plaque morphology, cardiac computed tomography, three-dimensional reconstruction

## Abstract

**Background:** Atherosclerotic plaques are the major cause of coronary artery disease (CAD). Currently, computed tomography (CT) is the most commonly applied imaging technique in the diagnosis of CAD. However, the accurate extraction of coronary plaque geometry from CT images is still challenging.

**Summary of Review:** In this review, we focused on the methods in recent studies on the CT-based coronary plaque extraction. According to the dimension of plaque extraction method, the studies were categorized into two-dimensional (2D) and three-dimensional (3D) ones. In each category, the studies were analyzed in terms of data, methods, and evaluation. We summarized the merits and limitations of current methods, as well as the future directions for efficient and accurate extraction of coronary plaques using CT imaging.

**Conclusion:** The methodological innovations are important for more accurate CT-based assessment of coronary plaques in clinical applications. The large-scale studies, de-blooming algorithms, more standardized datasets, and more detailed classification of non-calcified plaques could improve the accuracy of coronary plaque extraction from CT images. More multidimensional geometric parameters can be derived from the 3D geometry of coronary plaques. Additionally, machine learning and automatic 3D reconstruction could improve the efficiency of coronary plaque extraction in future studies.

## Introduction

With the increasing incidence, coronary artery disease (CAD) is the most common type of heart disease and the leading cause of death globally (1). The stenosis of coronary arteries incurred by the growth of atherosclerotic plaques is the major cause of CAD and related cardiac events such as acute myocardial infarctions (MI) (2). Therefore, the accurate evaluation of atherosclerotic plaques in coronary arteries is important for the diagnosis and treatment of CAD.

In the diagnosis of CAD, computerized tomography (CT) imaging is the most commonly used imaging technique. Cardiac or cardiovascular CT (CCT), also named as coronary computed tomography angiography (CCTA, sometimes short as coronary CTA), or CT coronary angiography (CTCA), has a high spatial resolution to reflect the anatomic severity and morphology of coronary plaques. The anatomic severity of coronary plaques estimated by CT imaging was in accordance with the results derived from intravascular ultrasound (IVUS) imaging (3). CT imaging has a higher resolution than the cardiac magnetic resonance imaging (MRI) (4). Compared with MRI and IVUS, CT is low-cost, non-invasive, and available on patients with implants (5). Furthermore, CT imaging could reflect the morphology of plaques by differentiating various compositions. Non-calcified, partially calcified, and calcified plaques could be differentiated based on their x-ray attenuation values (in Hounsfield units, or HU) which reflect the brightness of certain areas in CT images (6).

Since the early 2000s, the development of multi-slice CT (MSCT) technology, which refers to a special CT system equipped with a multiple-row (4, 8, 16, and 64) detector array that can collect a high volume of patient data in each gantry rotation, provides the possibility of reconstructing the three-dimensional (3D) geometry of atherosclerotic plaques in coronary arteries (7, 8). Especially, the 64-detector CT scanners showed better accuracy than the 4- or 8-detector ones in the diagnosis of significant coronary arterial stenosis (diameter stenosis >50%) (9). Based on the analysis of coronary CT images, the diameter stenosis and calcification volume have been widely used in the CAD-related clinical applications (10). In the meantime, the automatic 3D reconstruction and quantification of the non-calcified component was also achievable (11). The standardized, quantitative analysis of coronary CTA datasets was reproducible for the measurement of plaque geometrical and compositional parameters (e.g., plaque length, percentage area stenosis, and percentage of atheroma volume) in different geometric dimensions with high intra-observer and inter-observer agreement (12). Based on the comparison with histological images, the MSCT images have been applied in the analysis of coronary plaque morphology (13). Since the late 2000s, the 3D geometry of coronary plaque reconstructed from MSCT images has been widely applied in the computational simulation of plaque stress (14), wall shear stress (15), and the accumulation of low-density lipoprotein (16). The accurate extraction and reconstruction of coronary plaques from CT images plays a key role in improving the quality of diagnosis and treatment of CAD, as well as the pathophysiological studies of coronary arteries.

Currently, the majority of the studies on the extraction of coronary plaques from CT images are based on the difference in attenuation values, which is not sufficient for the accurate evaluation of coronary plaques. For calcified plaques, the blooming artifact could cause the overestimation of plaque areas, especially in the cases with significant calcification (17). For non-calcified plaque, it is difficult to differentiate between fibrotic and lipid plaques. Another challenge is the demarcation between the non-calcified or mixed plaques, the outer vessel border consisting of the tunica adventitia, and the surrounding tissues, which are similar in intensity (18). To achieve the accurate evaluation of coronary plaques using CT images, technical innovations are needed to overcome these challenges.

Recently, some novel methods and algorithms have been proposed to improve the accuracy of coronary plaque extraction from CT images. In this review, the novel methods and algorithms are categorized, analyzed, compared, and summarized to disclose the future directions toward more accurate CT-based evaluation of coronary plaques.

## Methods

The keywords for the literature search are “coronary artery” or “coronary” combined with “atherosclerotic plaque” or “plaque,” and “CT” or “computerized tomography”. Publications written in English from 2015 to June 2019 were searched on PubMed, Web of Science Core Collection, IEEE Xplore Digital Library, and https://scholar.google.com.

Over 50 papers have been found. Based on the titles, keywords, and abstracts, more than 20 papers were excluded which did not propose methodological or technological innovation of coronary plaque extraction. Finally, 31 papers were selected for the review including 27 journal articles and 4 conference papers.

The selected publications were categorized according to the dimension of the plaque extraction method. In the two-dimensional (2D) methods, the coronary arteries and plaques are directly segmented and extracted from the 2D images. In the 3D methods, the 3D structures of coronary arteries and plaques are reconstructed from the 2D images. At present, the clinical diagnosis and research of CAD are based on the CTCA images derived from the MSCT scan. The MSCT scan provides a solid basis for 3D CTCA analysis. Whereas, the original CT images derived from MSCT scans are still the transverse 2D images. The 3D CTCA images and the 2D CTCA images on coronal and sagittal cross-sections are obtained through reconstruction. As the original data, 2D CTCA images are essentially more accurate than other reconstructed images. Therefore, the 2D CTCA images are still widely used in some recent studies (19). The reconstructed 2D images, including the coronal, sagittal, and curved planar reformation images, also play an important role in clinical diagnosis (20). Additionally, the current diagnostic standards and guidelines are based on the geometric parameters (especially the diameter stenosis) derived from 2D images of coronary arteries (21). Therefore, the 2D methods are included and analyzed in this review while the 3D methods will be increasingly important in future studies.

Some studies included 3D reconstruction and volumetric measurement. However, in these studies, the 3D reconstruction was automatically performed by software without any technical details disclosed (22), and the aim was the comparison or validation of 2D image processing algorithms (23–27), without methodological innovation in 3D volumetric analysis (17). Therefore, they were categorized as 2D methods. Some studies used the automatic 3D reconstruction of coronary plaques, but volumetric measurement is a major objective (28–32), or the 2D images were extracted from 3D CTA images (33), therefore, they were classified as 3D studies.

In the following sections, 2D methods and 3D methods will be separately analyzed and summarized in three aspects: data, method, and evaluation. Regarding the data, we listed the details of data source (*in vivo, in vitro, ex vivo*, or phantom), inclusion criteria on arterial segment and plaques, numbers of human subjects and arterial segments. Regarding the method, we analyzed the classification of plaques (calcified and non-calcified; calcified, lipid and fibrotic; etc.), attenuation values of different plaques, methods of plaque extraction and reconstruction, as well as the technical innovations. Regarding the evaluation, we analyzed the geometric parameters in different dimensions, the intra- and inter-observer repeatability of the results, and the reference for the evaluation of accuracy (IVUS, histopathologic examination, etc.).

## Results

### 2D Methods of Coronary Plaque Extraction From CT Images

#### Classification of Data

We found 13 studies on 2D plaque extraction, including 12 original studies and a review paper (34). The majority (10 out of 12) of original studies used *in vivo* data which were collected non-invasively (17, 22–27, 35–37). Three studies used phantoms for data collection, in which 2 studies used phantom data in parallel with *in vivo* data (17, 37) while one used exclusively the phantom data (38). One study used *ex vivo* data (39).

In terms of the medical imaging techniques, *in vivo* data are the imaging data collected from living and functional organisms. *In vivo* imaging data are patient-specific. *In vivo* CT imaging data could be derived before and after the clinical treatment as the baseline and the follow-up observations to evaluate the severity of the CAD and the efficiency of treatment. Therefore, *in vivo* data play a key role in the diagnosis and treatment of CAD. However, CT scans are mainly performed on patients with CAD. *In vivo* data of healthy individuals are relatively rare.

CT imaging data could be collected from phantoms. A major benefit of phantoms is their controllable geometry. By presetting the geometric parameters (diameters, length, severity of stenosis, etc.) of plaque phantoms, and comparing with the geometry reconstructed from CT imaging, the accuracy of plaque extraction algorithms could be quantitatively evaluated. In a recent study, the accuracy of a vendor-specific model-based iterative reconstruction algorithm was evaluated on phantoms for both calcified and non-calcified plaques (37). A straight acrylic tube (length: 50 mm, diameter: 3 mm) was used as the model of coronary artery. Polystyrene, mono cast nylon, and acrylonitrile butadiene styrene copolymer were mixed to simulate soft, intermediate, and calcified plaques, with stenotic attenuation value of 40, 80, and 150 HU. Two stenotic degrees of 50% and 75% were used to evaluate the accuracy of plaque extraction in different plaques.

Using different plaque components, different anatomic structures, and different sizes, phantoms can be used in the comprehensive evaluation of plaque extraction methods. To optimize the CTCA protocol for more accurate extraction of plaques and coronary arteries, as well as early detection of the vulnerable plaques (non-obstructive atherosclerotic plaques with a thin fibrous cap covering fatty debris, leading to thrombus formation and embolization when ruptured), Kashani et al. used a phantom which contained 7 channels with different diameters between 3 and 5 mm. The channels were filled with different materials to simulate cholesterol and adipose tissues of the plaques, and surrounding myocardial tissues (38). The authors suggested that CTCA imaging of lipid-rich plaques can be optimized through using intermediate x-ray tube currents of 300 and 400 mA and the adjustment of the x-ray tube potential. To investigate the accuracy of iodine quantification with dual-energy CT imaging of coronary arteries, Pelgrim et al. developed an anthropomorphic phantom including artificial lungs, spine, body fat, and a cavity at the position of the heart. The cavity was filled with a holder carrying five separate tubes to simulate coronary arteries. Different patient sizes were simulated using extension rings with densities comparable to fat (40).

Another benefit of using phantoms is the avoidance of complex operation and clinical risks of *in vivo* imaging on human subjects or animals. To fully validate the results derived from phantoms, and investigate their clinical applications, two studies included both phantoms and *in vivo* data (17, 37). The first study focused on the calcified plaques with ≥50% and ≥70% luminal stenosis on CT images, and simulated them in phantoms to evaluate if the de-blooming algorithm would derive more accurate plaque extraction (17). The second study included both calcified and non-calcified plaques with 50% and 75% stenosis in a coronary vessel model whose length and radius were 50.0 and 3.0 mm, respectively (37).

Nevertheless, the use of phantoms also has some limitations. Firstly, phantoms have highly simplified geometry which could not present the patient-specific anatomy. It is commonly observed that the anatomy of human coronary arteries is highly diverse among the population. Secondly, it is difficult to use phantoms to simulate the mixed plaques consisting of lipid and fibrotic components with speckled calcifications (diameter <2 mm), which are widely observed especially in the early phase of calcification (41).

One study used *ex vivo* imaging data which were derived from three *ex vivo* human hearts during post-mortem. Both CCTA and IVUS imaging were performed. Coronary computed tomography angiography and IVUS images of arterial cross-sections in 1-mm increments were co-registered. To evaluate the accuracy of the proposed algorithm of plaque reconstruction, the plaque areas reconstructed from CCTA images were compared with the plaque areas in the corresponding IVUS images (39). The *ex vivo* imaging data could be desirable than *in vivo* data in some aspects. Firstly, imaging is much easier to perform on *ex vivo* specimen than on *in vivo* organ. Additionally, the *ex vivo* data are free from the motion artifact which is inevitable in *in-vivo* data. Therefore, *ex vivo* data could be used for quantitative evaluation of plaque reconstruction from patient-specific CT images. However, *ex vivo* imaging data were rare, therefore difficult to be used in large-scales studies.

#### Inclusion Criteria on Arterial Segments and Plaques

We included different types of articles covering scientific, engineering, and clinical studies. Normal subjects and CAD patients were recruited in different studies in which the inclusion (or exclusion) criteria of human subject are highly diverse. Therefore, we focused on the inclusion (or exclusion) criteria of arterial segments and plaques.

For arterial segments, due to the limited accuracy of CT imaging in small branches, some studies included only the arterial segments with a radius larger than 1.5 mm (22, 34). The inclusion of only the proximal 40 mm of each coronary artery and the exclusion of left main coronary lesions were also used as criteria (39). In three phantom studies, the first one used the inner diameter between 3.5 and 4.5 mm (17). The second one used the length of 50.0 mm and a diameter of 3.0 mm for lumen (37). The third one included lumen diameter between 3 and 5 mm, wall thickness between 1.5 and 3.5 mm, with 10 mm as the segment length (38).

The severity of luminal stenosis, defined as the ratio between lumen diameters in stenotic and normal segments, was widely used as the inclusion criterion of plaques, especially non-calcified plaques. In the studies on 2D plaque extraction, the severity of luminal stenosis varies between 25% and 75% (37). In clinical diagnosis, luminal stenosis larger than 50% is widely used as the criterion of significant stenosis (22, 35–37).

#### Numbers of Human Subjects and Arterial Segments

Most of the studies on 2D plaque extraction included <100 human subjects. However, multiple arterial segments can be extracted from the imaging data of one subject. Therefore, in some studies, there are more arterial segments than human subjects. As mentioned, the only study using *ex vivo* data included three *ex vivo* human hearts (39). The following paragraphs are focused on the studies using *in vivo* data.

There were 2 pilot studies, which included <10 human subjects (27, 37). In the first study, 12 arterial segments were extracted from the imaging data of 10 human participants (37). In the second study, to investigate the automatic extraction of both calcified and non-calcified plaques, three male patients with acute myocardial infarct were included. For each subject, three CCTA scans were performed with different imaging parameters. In each scan, 17 coronary segments were extracted following the coronary arterial model proposed by the American Heart Association (AHA) (27).

Two studies included 10–50 human subjects. The first one included 31 human participants as well as 2 phantoms, from which 375 coronary arterial segments and 77 calcified plaques were extracted (17). This study was focused on the reduction of blooming artifact in extracting calcified plaques; therefore, patients with non-calcified plaques were excluded. Another study included 43 subjects to investigate the derivation of coronary calcium scoring (CCS) from low-radiation-dose (24). The analysis was based on individuals; therefore, the number of arterial segments was not provided.

Six studies included more than 50 (range: 53–99) subjects. Rossi et al. extracted 144 stenosed segments of coronary arteries from 99 patients to investigate if CTCA could be used in screening the functionally significant coronary lesions (35). To investigate the semi-automatic coronary plaque quantification, Øvrehus et al. collected the CTA data of 50 patients in which 627 arterial segments were confirmed as evaluable (diameter > 1.5 mm, without motion artifact) by observers. Luminal stenosis of > 70% and 50–70% was found in 1 and 4 patients, respectively. Non-calcified and mixed plaques were found in 17 and 55 arterial segments (22). Li et al. proposed a new algorithm to improve the accuracy of reconstructing non-calcified plaques (36). Seventy-seven non-calcified plaques were extracted from 66 patients. The analysis was plaque-based therefore the number of arterial segments was not disclosed (36). Similarly, two studies investigated the reconstruction of calcified plaques based on Agatston coronary artery calcium scoring (CAC) scoring, with CT data collected from 63 (23) to 60 (25) subjects, respectively, without mentioning the number of arterial segments. Another study investigated plaque compositions divided by five ranges of HU values. Totally 160 plaques were extracted from 53 patients, without mentioning the number of arterial segments (26).

In addition, there is a clinical literature review which includes over 6,000 cases (34). This large and diverse population was used to cover a wide range of vulnerable and non-vulnerable plaques, which differ in severity and composition.

Considering the individual differences in the geometry of coronary arteries and plaques, recruiting large numbers of subjects could provide enough data for reliable statistical analysis. However, some of the presented studies have a small number of participants as they are pilot studies or phantom-based validation of algorithms. The extraction of multiple arterial segments from one subject is an important method to enlarge the sample size of arteries.

#### Classification of Coronary Plaques

The development of atherosclerosis starts from the filtration of low-density lipoprotein through the endothelium, which forms the fatty streaks in the arterial wall. The consequent inflammatory response involves macrophages, T-cells, and complex biochemical mechanisms, forming lipid-rich atherosclerotic plaques which finally become calcified and fibrous. According to the criteria proposed by AHA, the development of atherosclerotic plaques in coronary arteries consists of eight major stages: 1. Isolated macrophage foam cells; 2. Multiple foam cell layers formed; 3. Isolated extracellular lipid pools added; 4. Confluent extracellular lipid core formed; 5. Fibromuscular tissue layers produced; 6. Surface defect, hematoma, and thrombosis; 7. Calcification predominates; and 8. Fibrous tissue changes predominate (42).

Based on the AHA criteria, in grating-based phase-contrast computed-tomography (gb-PCCT) imaging of coronary arteries, four types of plaques could be differentiated: 1. Plaque with lipid or necrotic core surrounded by fibrous tissue with possible calcification; 2. Complex plaque with possible surface defect, hemorrhage or thrombus; 3. Calcified plaque; and 4. Fibrotic plaque without lipid core and with possible small calcification (43). In the histological analysis, the calcified (44), fibrotic (or fibrous), and lipid (or lipid-rich) plaques (45) can be easily differentiated by the image features (Table 1). In general CT imaging, atherosclerotic plaques in coronary arteries can be classified into three categories: calcified, non-calcified, and partially calcified (or mixed) (50). Based on the advanced analysis of CT images, the non-calcified plaques could be further classified as lipid and fibrotic plaques (46), as illustrated in Table 1, which has been applied in 2D plaque extraction (26). Different classification methods of coronary plaques have been summarized in Table 2. Considering the difference between studies in the resolution of CT imaging, the accurate separation between lipid-rich and fibrotic plaques remains challenging (50). In clinical CT imaging, calcified and fibrotic plaques are frequently observed in fully developed stenoses since they reflect the late stages in the progression of atherosclerosis. Whereas, lipid-rich plaques are less commonly observed since they mainly represent the initial stage of atherosclerosis, when the plaque could not cause severe stenosis and hemodynamic effects.

**Table 1:** Graphical representation of described examples of calcified and non-calcified plaques.

**Methodology**	**Calcified plaques**	**Non-calcified plaques**	
		**Fibrotic**	**Lipid**
Histological sample	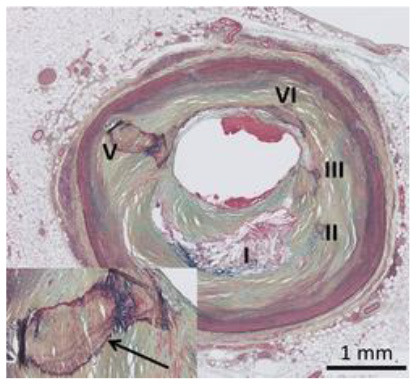	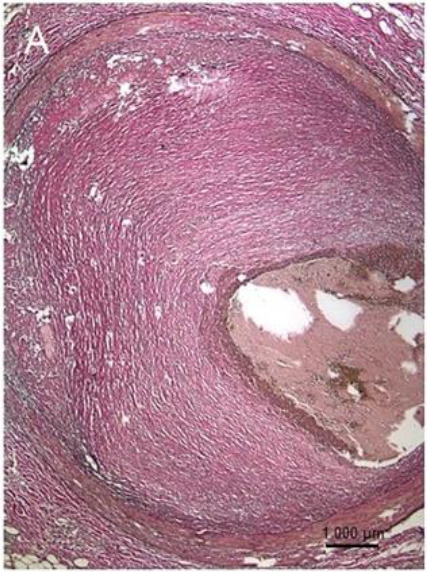	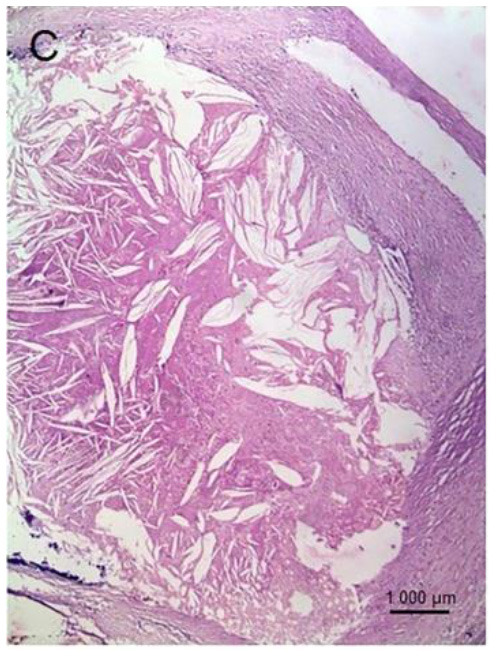
	I: late fibro-atheroma (LFA) lesion (necrotic core covered by a fibrous cap). II-V: consolidated former lesions [fibrotic calcified plaque (FCP)]. Adapted from Lindeman et al. (44) 2018 by the authors. CC BY 4.0.	Fibrous plaque, which is fibrocellular and also rich in proteoglycans (Elastic van Gieson, 100) Adapted from Vaideeswar et al. (45) 2019 by the authors. CC BY 4.0.	Fatty plaque, comprising a large lipid-rich core separated from the lumen by a thin fibrous cap. The lipid material may in the form of collections of foamy macrophages and/or extra-cellular lipid material (HE, 100). Adapted from Vaideeswar et al. (45) 2019 by the authors. CC BY 4.0.
2D plaque reconstruction	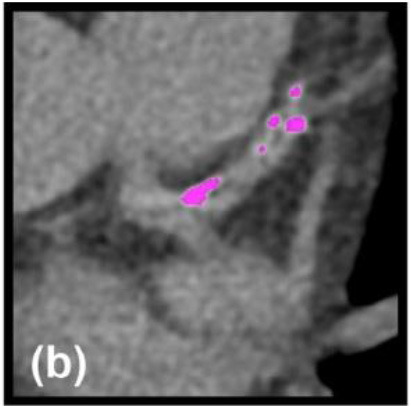	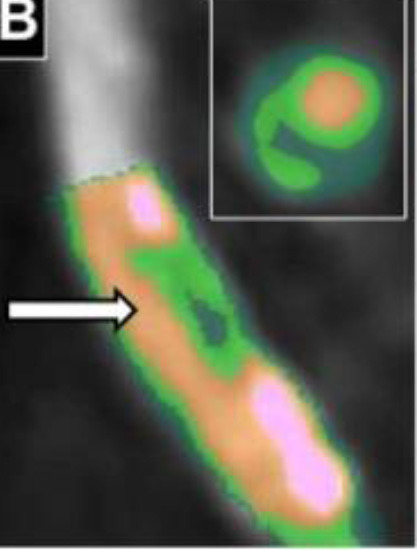	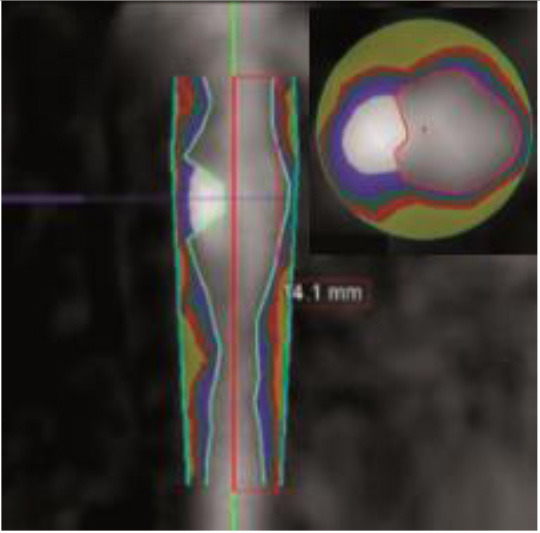
	Calcification marked with purple mask. Adapted from Messerli et al. (25) 2016 with permission from The Association of University Radiologists.	The necrotic core (35.3%, dark green) surrounded by fibrous plaque (51.5%, light green). Lumen and calcification (13.2%) are marked orange and pink, respectively. Inset shows cross-sectional images at arrows. Adapted from Obaid et al. (46) 2017 by the authors. CC BY 4.0.	A lipid plaque: yellow <51 HU, red 51–100 HU, green 101–150 HU, blue 151–350 HU, white >350 HU. Adapted from Chen et al. (26) 2016 with permission from The Foundation Acta Radiologica.
3D plaque reconstruction	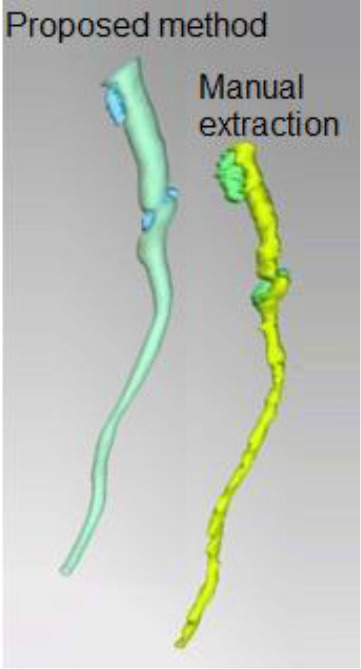	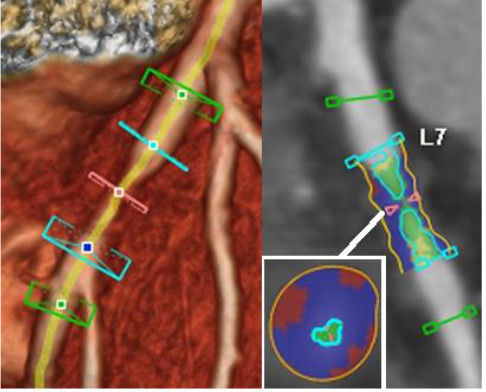	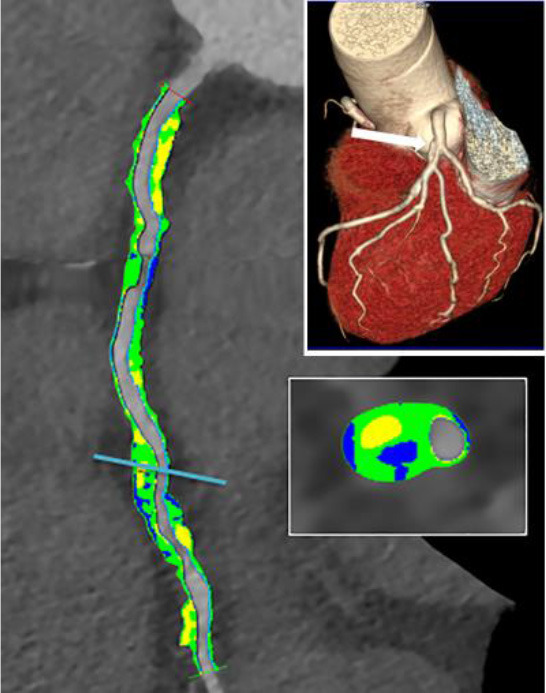
	Lumen and calcified plaque reconstructed by semi-automatic and manual methods. Adapted from Kigka et al. (47) 2017 with permission from Elsevier Ltd.	The semi-automatic analysis of a fibrotic plaque in LAD: lipid (red), fibrous (blue) and calcified (yellow) components. Adapted from Cui et al. (48) 2017 by the authors. CC BY 4.0.	Semi-automatic segmentation of coronary vessel: calcified (yellow), lipid (green) and fibrotic (blue). The cross-section shows a lipid plaque. Adapted from Infante et al. (49) 2019 by the authors. CC BY 4.0.

**Table 2:** Methods of coronary plaque classification.

	**Plaque classification**		
	**Calcified and non-calcified**	**Calcified, mixed, and non-calcified**	**Lipid-rich, fibrous, and calcified**
2D reconstruction	Calcified and non-calcified plaques (22, 27, 35, 39). Calcified and non-calcified plaques, as well as high-risk plaques. The main CTCA features related to high-risk plaques are: (1) positive remodeling, PR [remodeling index (RI) ≥1.1]; (2) low-attenuation plaque, LAP (<30 HU); (3) napkin-ring sign, NRS (description below); and (4) spotty calcifications, SCs (<3 mm) (34).		Different plaque combinations (soft, intermediate, and calcified), different stenosis (50% and 75%), different lumen densities (low and high lumen), positive remodeling and spotty calcium (37). Non-calcified plaques, including fibrous and lipid-rich ones, components segmented using different thresholds: <51, 51–100, 101–150, 151–350, and >350 HU intervals (26).
3D reconstruction	Calcified and non-calcified plaques (32, 47, 51, 52).	Calcified plaque, non-calcified plaque, and mixed plaque, i.e., a plaque containing calcified and non-calcified components (53). Mixed (51%), non-calcified (31%), and calcified (18%) plaques (54). 729 non-calcified plaques, 511 calcified plaques, and 546 mixed plaques (33). The plaque area was stratified into <60 HU (lipid-rich plaque) and >180 HU (calcified plaque) parts (29). Soft lipid-rich plaques, mixed plaques, calcified plaques (55).	Lipid, fibrous, and calcified components as <60 HU, 60–200 HU, and > 200 HU (31). Lipid-rich, fibrotic, and calcified plaques (28). Non-calcified plaque, low-density non-calcified plaque, and calcified plaque (56). Low-density non-calcified plaques (different thresholds of attenuation values used) and calcified plaques (30).

In the 13 studies on 2D plaque extraction, 4 studies included only calcified plaques (17, 23–25). Two studies were mainly focused on non-calcified coronary plaques (36, 38). Seven papers included both calcified and non-calcified coronary plaques, which gives us an overview of the progression of atherosclerosis in CAD patients (22, 26, 27, 34, 35, 37, 39).

#### Attenuation Values of Different Plaques

The attenuation value of calcification is much higher than that of the surrounding tissues. Therefore, the differentiation of calcified and non-calcified plaques on CT images could be achieved by setting thresholds of attenuation (24). The threshold for calcified plaques varies in different studies (see Table 3). An earlier study in 2011 used the ranges of 30–70 HU for non-calcified plaques and >70 HU for calcified plaques (57). Another study in 2014 used the ranges of −10–69, 70–129, and >400 HU to differentiate lipid, fibrotic, and calcified plaques (58).

**Table 3:** Comparison of attenuation values used in selected studies.

**Study**	**Year**	**Dimension of reconstruction**	**Attenuation values (in HU units)**	
			**Calcified plaques**	**Non-calcified plaques**
Li et al. (36)	2019	2	N/A	<60 for low-attenuation plaques <200 for high-attenuation rim with an inner hypo-attenuation area (<130)
Li et al. (17)	2018	2	1097–2910	N/A
Funama et al. (37)	2017	2	150	40 for soft plaques80 for intermediate plaques
Braber et al. (24)	2016	2	>130 for Agatston Scores >351 and >600 for plaque reclassification	N/A
Messerli et al. (25)	2016	2	>130	N/A
Rodriguez-Granillo et al. (34)	2016	2	>130	<30 for low-attenuation plaques
Matsumoto et al. (30)	2019	3	>500	<150 for non-calcified plaques <30 and <45 for lipid-rich plaques
Kigka et al. (47)	2018	3	>400	<50
Wang et al. (31)	2017	3	>200	<60 for lipid plaques60–200 for fibrotic plaques
You et al. (28)	2016	3	≥130	0–49 for lipid plaques50–129 for fibrotic plaques
Puchner et al. (29)	2015	3	>180	<60

Attenuation scale has been used to differentiate non-calcified coronary plaques (59). However, the differentiation of non-calcified plaques is difficult due to the limited contrast between the fibrotic and lipid tissues in attenuation value (Table 3). Additionally, the attenuation values of a non-calcified plaque and its neighboring tissues are not significantly different around the boundary. Therefore, it is difficult to develop the fully automatic methods to reconstruct the non-calcified plaques and differentiate the components of non-calcified plaques.

For calcified plaques, Agatston CAC scoring is widely used as an estimation of the total amount of calcium for the prediction of adverse cardiovascular events in people with CAD. It is calculated using high calcium area slice of the CT image, multiplied by the maximal attenuation of the calcification in individual case (60). The attenuation value qualitatively reflects the different types (calcified and non-calcified) of plaques whereas the Agatston score quantitatively represents the calcification in coronary arteries.

#### Methods of Plaque Extraction and Reconstruction

The methods of 2D plaque extraction can be categorized as semi-automatic and automatic. During extraction, manual interaction is indispensable in semi-automatic methods but is infrequent in automatic methods. The manual interactions include: setting the boundaries of areas for analysis such as the start and end points of arterial segments and plaques, adjusting the automatic segmentation and extraction results to revise the geometric errors, and using the manual extraction results to train or validate new algorithms. Other manual interactions include setting parameters in image processing and selecting CT images for analysis. The details are listed in Table 4.

**Table 4:** Manual manipulations in automatic and semi-automatic methods of plaque extraction.

		**Study**	**With manual manipulation**				
			**Set the boundary of analysis**	**Adjust the result when needed**	**Generate results to train the algorithm**	**Generate results to validate the algorithm**	**Others**
2D reconstruction	Semi-automatic	Messerli et al. (25)	√				
		Rossi et al. (35)	√	√		√	
		Precht et al. (27)	√	√			
		Øvrehus et al. (22)	√				Window level and width of image reconstruction were adjusted for optimal visualization at the discretion of the observer.
		Szilveszter et al. (23)	√				
		Chen et al. (26)	√				
		Braber et al. (24)		√			
	Automatic	Li et al. (17)				√	Optimal cardiac phase with the least motion artifact was selected manually by the operator.
		Li et al. (36)				√	
		Funama et al. (37) (not explicitly pointed out as automatic)				√	
		Kashani et al. (38) (not explicitly pointed out as automatic)	√				
		Puchner et al. (39) (comparison between automatic and semi-automatic algorithms)		√			
3D reconstruction	Semi-automatic	Kigka et al. (47)				√	
		Matsumoto et al. (30)	√			√(extract IVUS images)	Co-registration of IVUS and CTA images
		Sakellarios et al. (52)			√		
		Gaur et al. (56)	√				
		Puchner et al. (29)		√			
		Wang et al. (31)		√			
		You et al. (28)		√			
		Sun et al. (61)		√			
		Athanasiou et al. (62)			√	√	Co-registration of IVUS and CTA images
		Wei et al. (63)	√		√	√	
	Automatic	Jawaid et al. (64)				√	
		Ghanem et al. (55)				√	
		Károlyi et al. (32)	√				
		Zhao et al. (33)	√			√	Using Rotterdam Coronary Artery Evaluation Dataset
		Zreik et al. (53)			√	√	
		Kang et al. (51)			√	√	
		Park et al. (54) (comparison between automatic and semi-automatic algorithms)		√		√	

Messerli et al. proposed a semi-automatic method to evaluate CAC on software (syngo.via CT CaScoring, Siemens Healthcare) (25). Firstly, coronary lesions with attenuation >130 HU were automatically color-coded. Then the calcified coronary structures were manually selected. Finally, the software automatically calculated the Agatston score, CAC volume (mm^3^), and CAC mass (mg/cm^3^). Similarly, Szilveszter et al. (23) used software to identify the coronary artery plaques with area ≥1 mm^2^ and density >130 HU. Coronary plaques were selected manually to enable the semi-automatic software to calculate CAC scores.

The manual segmentation by tracing the proximal and distal plaque boundaries (26), and the visual examination and manual adjustment (24) were common in semi-automatic methods. Øvrehus et al. used manual interactions in imaging reconstruction and plaque segmentation (22). In multiplanar reformats of CTA images, a circular region of interest was placed in the aorta to define the “normal reference bloodpool.” The proximal and distal boundaries of each lesion were identified and marked by the reader. The software then automatically tracked the centerline of the coronary artery and quantitatively analyzed the plaques. Rossi et al. proposed a semi-automatic method to compare the visual and quantitative evaluations of plaques in CTCA images, in which various manual interactions were involved (35). Firstly, the CTCA data sets were evaluated visually, and the coronary lesion was graded as non-obstructive (<50% lumen narrowing), moderate (50% ≤ lumen narrowing <70%), and severe (≥70% lumen narrowing). Afterwards, the proximal and distal endpoints of coronary vessels with lumen diameter reduction ≥30% were manually marked. The lumen and vessel borders were generated automatically and adjusted by an experienced observer. The quantitative analysis of plaques was automatically completed by software. Precht et al. adopted similar semi-automatic method to estimate plaque volume in low-dose CCTA (27). The centerlines of the coronary arteries were automatically extracted and manually corrected when needed. The extracted arteries were manually partitioned according to the AHA 17-segment model. For each artery, the contours of lumen and outer vessel wall were automatically detected and manually fine-tuned by two independent observers with more than 7 years of experience. The manual fine-tuning of the automatic contour detection only showed a 0–7.3% deviation which did not significantly influence the final results. Puchner et al.'s compared the automatic and semi-automatic methods in generating vessel wall boundaries (39). The boundaries of vessel wall (inner, outer, or both) were manually corrected.

The manual segmentation results play an important role in the validation of automatic algorithms. Li et al. developed an automatic algorithm for CT image processing (36). For validation, two experienced radiologists independently identified plaque characteristics on the images reconstructed with different algorithms. Similarly, Funama et al. used the consensus of two CT image reviewers in plaque evaluation to compare different image processing algorithms (37). The reconstructed CT images were manually classified into four levels of quality, in vessel and plaque areas, respectively. Li et al. proposed an automatic de-blooming algorithm (17). Coronary computed tomography angiography images of phantoms were manually selected by the operator to find the images at the optimal cardiac phase with the least motion artifact. For *in vivo* CCTA images, an experienced reader independently reviewed all data sets, noted coronary calcification, and measured the volume of calcified plaques, coronary diameter stenosis (%), as well as the coronary area stenosis (%) on software as the reference for validation. Kashani et al. optimized the parameters of CCTA by comparing the quality of images derived by different parameter values (38). The contrast-to-noise ratio (CNR) was measured manually by prescribing a 0.018–0.021 cm^2^ region of interest in the center of the plaque and pericoronary fat in 8 different locations.

For 2D plaque extraction, there are more semi-automatic methods than automatic methods. Automatic methods are efficient and convenient for the large-scale extraction of coronary arteries and plaques from the CT images. However, the plaque size is often over- or underestimated when using automatic software. Hence, the manual interactions including editing and analysis are useful to improve the accuracy of 2D plaque extraction (22).

#### Technical Innovations

Filtered back projection (FBP) is a traditional type of algorithm that can indicate an attenuation value to each pixel (65) on CTA images. Filtered back projection reconstruction assumes that each pixel accurately indicates the attenuation. In 2D coronary plaque reconstruction, FBP is widely used to derive the reference values for the validation of new algorithms. Iterative reconstruction (IR) algorithms have the potential to improve the quality of CT image by reducing image noises and blooming artifacts when compared with FBP. IR algorithms are the majority of new algorithms in 2D coronary plaque reconstruction. In the following paragraphs, the algorithmic innovations will be summarized in terms of the improvement of accuracy in extracting different types of plaques.

##### Calcified Plaques

Calcified plaques are easy to extract from CT images due to their high attenuation values. Nevertheless, the brightness of a calcified plaque could affect its neighboring pixels by increasing their attenuation values. Consequently, the overestimation of the calcification size is common. Li et al. developed a de-blooming algorithm and applied it on CCTA images of 31 patients (17). They found that the de-blooming algorithm reduced the calcification volume and the stenosis in diameter by 48.1 ± 10.3% 52.4 ± 24.2%, respectively. However, the details of this algorithm were not disclosed (17).

Szilveszter et al. investigated the impact of iterative model reconstruction (IMR) on coronary artery calcium quantification as compared with the standard FBP and hybrid iterative reconstruction (HIR) algorithms (23). CT images of 63 individuals were reconstructed with FBP, HIR, and IMR. HIR and IMR resulted in lower CAC scores as compared with FBP (both *p* < 0.001). There was no difference between HIR and IMR (*p* = 0.855). The authors concluded that the utilization of IMR for CAC scoring can reduce the measured calcium quantity (23).

Braber et al. investigated the quantification of calcification in low-dose CCTA images using IR (24). Coronary artery calcium was quantified with Agatston scores on the CCS images using a semi-automatic software package (HeartBeat-CS; Philips Healthcare, Best, the Netherlands). For Agatston scoring, CAC was defined as regions with ≥130 HU within coronary arteries. All regions with density higher than 130 HU were automatically indicated by the software package. Calcification volumes were derived with a semi-automatic software package (QAngio CT v2; Medis Medical Imaging Systems, Leiden, the Netherlands) (24).

However, it was also suggested that IR could underestimate the calcification. Messerli et al. evaluated the influence of advanced modeled iterative reconstruction (ADMIRE) on the coronary artery calcium (CAC) scores, with FBP algorithm as the reference (25). CT images of 60 patients were reconstructed with FBP and ADMIRE at incremental strength levels of 1, 2, 3, 4, and 5, resulting in a total of 6 datasets. In four patients with low calcium burden, the use of ADMIRE 2 or higher resulted in the disappearance of calcium that was detectable using FBP. The authors concluded that ADMIRE causes a substantial reduction of the CAC scores measured by cardiac CT, which leads to an underestimation of cardiovascular risk scores in some patients (25).

##### Non-calcified Plaques

As to non-calcified plaques, the contrast in grayscale values between plaques, arterial walls, and surrounding tissues is low in CT images. The attenuation values or CAC/Agatston scales could not reflect the exact geometry of non-calcified plaques. To accurately separate the non-calcified plaques from the surrounding tissues, the morphological properties of non-calcified plaques need to be considered. Therefore, new algorithms based on IR (23) have been proposed to improve the quality of CT image in order to find the morphological and geometrical properties of the boundary between non-calcified plaques and surrounding tissues (17).

Li et al. assessed the effects of IMR algorithm on image quality in demonstrating the characteristics of high-risk non-calcified plaques in coronary arteries, in comparison with the HIR algorithm (36). The 256-slice CT images were derived from 66 patients with 77 non-calcified plaques. Paired CT image sets were reconstructed by HIR and IMR, respectively, on which plaque characteristics were compared. The signal-to-noise ratio (SNR) and CNR of the images, as well as the CNR between the plaque and adjacent adipose tissue, were also compared between the two reformatted methods. The napkin-ring sign appeared in 40 and 19 plaques reconstructed with IMR and HIR, respectively, which are significantly different (*p* < 0.001). Compared with HIR, IMR derived lower image noise (10 ± 2 HU vs. 12 ± 2 HU; *p* < 0.01), higher SNR and CNR, and especially higher CNR between plaques and surrounding adipose tissues (*p* < 0.01). The authors concluded that IMR can significantly improve image quality compared with HIR for the demonstration of atherosclerotic plaques in coronary arteries (36).

Furthermore, Chen at al. applied IR algorithm in the assessment of plaque vulnerability (26). They compared coronary plaque volume and low attenuation (lipid-rich) component derived by IR and FBP, respectively, from CTA images of 53 patients. Coronary plaques were identified by a board-certified radiologist (14 years of experience in cardiac CT). Then post-processing was performed by a research assistant trained in plaque volumetric analysis. The analysis was done in multiplanar reformat (MPR) using a semi-automated software (Aquarius iNtuition 4.4.6, TeraRecon Inc., Foster City, CA, USA). Proximal and distal plaque boundaries were traced manually. Total plaque volume was then obtained automatically. Plaque composition was assessed using attenuation (HU) intervals. It was found that IR significantly decreased the noise and increased SNR and CNR compared with FBP. Plaque characterization was performed in 41 patients for a total of 125 plaques. Regarding the total plaque volume and the low attenuation plaque component, there was no statistically significant difference between all IR levels and FBP. The authors concluded that no significant impact on plaque vulnerability assessment should be expected when using IR vs. FBP for plaque reconstruction from CTA images (26).

##### Different Plaques

Puchner et al. applied IR algorithm in semi-automated extraction of different plaques (fibrous, fatty, or fibrofatty, and the presence of calcification) (39). Coronary computed tomography angiography and IVUS images of seven coronary arteries were acquired *ex vivo*. Images of 173 cross-sections of coronary arteries were coregistered between CCTA and IVUS in 1-mm increments. Coronary computed tomography angiography images were reconstructed using FBP with adaptive statistical (ASIR), and model-based (MBIR) iterative reconstruction algorithms. Fully automated (without manual corrections) and semi-automated (allowing manual corrections of vessel wall boundaries) plaque burden assessments were performed for each reconstruction algorithm. Agreement between CCTA results and IVUS was evaluated with Pearson correlation. It was found that manual correction of the semi-automated assessments improved plaque burden correlation with the IVUS assessment independently of reconstruction algorithm (*p* < 0.0001). Furthermore, MBIR was superior to FBP and ASIR in semi-automated and fully automated plaque extraction (all *p* < 0.001). It was concluded that MBIR with semi-automated assessment could improve the accuracy of plaque burden assessment in CCTA images (39).

##### Overall Plaque Burden

Precht et al. compared ASIR and MBIR reconstruction algorithms on quantitative measurements of plaque volumes and intensities in coronary arteries (27). Dose-reduced CCTA were derived from 3 patients and reconstructed with 30% ASIR (CTDIvol at 6.7 mGy), 60% ASIR (CTDIvol 4.3 mGy) and MBIR (CTDIvol at 1.9 mGy). Quantitative coronary plaque analysis was performed. Centerlines of the coronary arteries were automatically extracted and manually corrected. The extracted vessels were manually partitioned according to the AHA 17-segment model. The contours of lumen and outer vessel wall were automatically detected and manually fine-tuned. The plaque burden was calculated as the ratio between total plaque volume and total vessel volume. It was found that plaque volume and plaque burden show a decreasing tendency from ASIR to MBIR. The lumen and vessel volume decrease slightly from 30% ASIR to 60% ASIR. The intensities did not change overall between the ASIR and MBIR reconstructions for either lumen or plaque (27).

Funama et al. investigated the effect of contrast enhancement on the stabilities of plaque attenuation, using FBP and IR algorithms in imaging reconstruction (37). 320-detector volume scanning was performed on phantoms of vessel tubes with stenosis and a tube without stenosis using three types of plaque attenuation values. CTA images were reconstructed with FBP and two types of IR [AIDR3D and FIRST (forward-projected model-based iterative reconstruction solution)], with stenotic attenuation value of ~40, 80, and 150 HU, respectively. In each case, the tubing of the coronary vessel was filled with diluted contrast material and distilled water to reach the target lumen attenuation values of ~350, 450, and 0 HU, respectively. It was found that at 50% stenosis, the plaque attenuation value with contrast enhancement increased for FBP and AIDR3D, and the difference in the plaque attenuation value with and without contrast enhancement was 15–44 HU for FBP and 10–31 HU for AIDR3D. However, the plaque attenuation value for FIRST had a smaller variation and the difference with and without contrast enhancement was −12–8 HU. The validation study was performed on CT images of 10 patients where FIRST derived the highest CNR in vessels and plaques. The authors concluded that the FIRST method improves the visualization of coronary plaques in coronary CT angiography (37).

#### Geometric Parameters in Measurement

In 2D reconstruction, some geometric parameters could be directly measured from the 2D images, including cross-sectional area, lesion length, minimal area diameter, and mean vessel size for the affected blood vessels (39). These parameters reflect the size of plaques and affected arterial segments.

The extent of coronary plaque could also be quantitatively evaluated by plaque burden, which is defined as the percentage of plaque in cross-section area: PB = (ACN-ACS)/ACS, where PB denotes plaque burden while ACN and ACS denote the cross-section areas in normal and stenotic arterial segments, respectively (39, 66).

#### Intra- and Inter-Observer Repeatability

Intra-observer and inter-observer repeatability reflect the consistency between repeated measurements performed by one observer and different observers, respectively. The expertise in coronary CT imaging and diagnosis can improve the intra- and inter-observer repeatability (67). Acquiring expertise in CTA interpretation may take more than a year. It has been reported that, for coronary CT imaging, the intra- and inter-observer repeatability on plaque volume estimation depends on the size of plaque (68). Furthermore, the repeatability results indicated that the percentage of plaque composition is more reliable than plaque volume (69). Therefore, the estimation of intra- and inter-observer repeatability is important to validate the reliability of plaque extraction methods.

In the 13 studies on 2D plaque extraction, the intra-observer repeatability was evaluated in five studies (22–24, 26, 38). Inter-observer repeatability was evaluated in 7 studies, (17, 22, 23, 26, 27, 36, 37). Six studies reported that the measurements were repeated by at least one expert with more than 5 years of experience (17, 26, 27, 36, 37). Comparatively, the experience of expert was between 1 and 5 years in other two studies (22, 23).

#### Reference of Accuracy

To evaluate the accuracy of plaque extraction algorithms, the reconstructed coronary plaques from 2D CT images were often compared with the results derived from invasive coronary angiography (ICA) (35), IVUS (39), or pre-defined geometric parameters in phantoms (38). Compared with CT imaging, the ICA and IVUS are more accurate in reflecting the geometric details of lumen, therefore have been widely used as the reference in related studies (70, 71).

### 3D Methods of Coronary Plaque Extraction From CT Images

#### Classification of Data

We found 17 papers focused on the 3D methods of coronary plaques extraction and reconstruction, including 16 original studies (28–33, 47, 51–56, 61, 62, 64), and a review paper (72). The same method was adopted in Athanasiou et al. (73) and Sakellarios et al. (52). The imaging data used in 3D plaque extraction include phantom, *ex vivo, in vitro*, and *in vivo* data.

A study used 17 plaque phantoms in three different types of attenuation, to investigate the reliability of low radiation dose CT imaging in representing the 3D geometry of plaques. By using phantoms, the accuracy of plaque extraction could be quantitatively evaluated in volume (31).

In 3D plaque extraction, *ex vivo* imaging data enable the researchers to perform accurate 3D geometric measurements (29). The results of plaque extraction could be compared with the histopathological measurement as the ground truth (29). Nevertheless, the lumen of an *ex vivo* artery is hollow whereas the lumen of the corresponding *in vivo* artery is filled with blood which has cyclic changes in pressure, velocity, and wall shear stress. Consequently, considering the effects of cyclic fluctuations of blood flow on the deformation and mechanical properties of arterial walls, the geometry of *ex vivo* arteries could be different from *in vivo* ones, which is a major limitation of *ex vivo* data (74).

*In vitro* data, here defined as the imaging data derived from patient-specific models of coronary arteries, could provide patient-specific geometry parameters of plaques and affected arteries. Compared with *in vivo* and *ex vivo* data, *in vitro* data could accurately reflect the geometry of arteries without motion artifact. Sun et al. used *in vitro* data of calcified plaques with different severities from three subjects to investigate the effect of slice thickness and beam energy on the accuracy of synchrotron radiation CT imaging. The *in vitro* arterial models were generated from the original high-resolution CCTA images using 3D printing technique. The plaques reconstructed from the synchrotron radiation CT images of the *in vitro* models were compared with those reconstructed from the origin CCTA images (61). Using *in vitro* data, the accuracy of plaque extraction could be quantitatively evaluated in anatomical details of patient-specific geometry. However, the high-resolution images for *in vitro* model reconstruction, the materials and devices for 3D printing, as well as the multiple imaging operations for comparison, limited the further application of *in vitro* data in clinical diagnosis.

*In vivo* data were used in the majority (14 out of 17) of the original studies on 3D plaque extraction (28, 30, 32, 33, 47, 51–56, 62, 64) and was mentioned in the review paper [(63) in (72)]. With 3D reconstruction, the diversity in the geometry and composition of plaques could be fully disclosed and represented not only on cross-sections but also in the longitudinal direction along the vessel, as well as in volume. Therefore, *in vivo* data play a key role in investigating the 3D geometry and composition of coronary plaques.

#### Inclusion Criteria on Arterial Segments and Plaques

The inclusion criteria in 3D plaque extraction studies are more diverse compared with 2D studies. In 3D reconstruction, the geometric details in different dimensions and the combination with plaque composition generated more detailed inclusion criteria for arterial segments and plaques compared with 2D reconstruction.

For arterial segments, the diameter (28) [as summarized in (72)] and length of segment (56, 64) were widely used as in 2D reconstruction studies. In a study on the prediction of all-cause mortality in CAD patients based on CCTA images (55), 16 arterial segments were extracted and 3D reconstructed according to a 16-segment model of coronary arterial tree (75). In another study, to investigate the difference between proximal and distal segments of the main coronary arteries [anterior descending artery (LAD), left circumflex artery (LCX), right coronary artery (RCA)], the middle segments and side branches were excluded (32). In some studies, arterial segments especially distal branches were excluded due to low quality of images (30, 54).

Regarding the coronary plaques, the severity of included stenoses ranged from 25% to more than 90% (47, 51). More detailed inclusion criteria have been proposed based on the analysis of plaque geometry in different dimensions. Gaur et al. investigated the difference between calcified and non-calcified plaques in FFR, where the criterion of spotty calcification was defined as visually identified calcifications comprising <90° of the vessel circumference and <3 mm in length (56). Similarly, in another study which investigated the accuracy of 3D reconstruction of lipid-core plaques, a lipid-core plaque was defined as any fibroatheroma with a lipid core >60° in circumferential extent, with a core width of >200 μm and a cap thickness of <450 μm (29).

#### Numbers of Human Subjects and Arterial Segments

The number of human subjects covers a wide range (3–254) in different studies on 3D reconstruction of coronary plaques. Firstly, only three human subjects were included in the two pilot studies using exclusively *ex vivo* and *in vitro* data (29, 61). In contrast, the studies using *in vivo* data included at least 8 human subjects (Table 5). Secondly, regarding the studies using *in vivo* data, those included <30 human subjects were focused on algorithmic development with limited validation (52, 64) or initial validation (47, 62, 73). In contrast, the studies including more than 80 subjects were aimed for the full validation of existing algorithm (63), the pathological indication of results (28), and clinical applications (54, 56).

**Table 5:** Characters of data in the studies of 3D coronary plaque analysis.

**Study**	**Year**	**Type of data**	**Number of subjects**	**Number of stenosis or sections**	**Standards of inclusion or exclusion of artery segment (length of diameter)**
Gaur et al. (56)	2016	*in vivo*	254	484 vessels	Coronary segments ≥2 mm with plaque were analyzed. Spotty calcification was visually identified as calcifications comprising <90° of the vessel circumference and <3 mm in length
Jawaid et al. (64)	2018	*in-vivo*	8	13 non-calcified segments	Maximum arterial segment length of 6 mm used for segmentation
Sakellarios et al. (52)Athanasiou et al. (73)	2016	*in-vivo*	20	20	N/A
You et al. (28)	2016	*in-vivo*	87	N/A	diameter >2.0 mm
Ghanem et al. (55)	2019	*in-vivo*	41	122 plaques	16 arterial segments in each case, according to a modified coronary arterial tree model (75).
Wei et al. (63) as summarized in the review by Jawaid et al. (72)	2018	*in-vivo*	83	120 soft plaques	Radius <3 mm to ensure that whole arterial cross-section is covered since 2.5 mm is the maximum radius of coronary arteries
Athanasiou et al. (62)	2016	*in-vivo*	10	8 calcified plaques deposits	N/A
Park et al. (54)	2015	*in-vivo*	142	150 coronary artery segments	11 segments excluded due to the insufficient IVUS or CT image quality (seven segments) caused by severe calcification or motion artifacts
Kigka et al. (47)	2018	*in-vivo*	12	12 arteries	N/A
Károlyi et al. (32)	2017	*in-vivo*	52	468 segments, with 41 calcified or partially calcified plaques identified	proximal and distal coronary segments, with middle coronary segments and side branches excluded
Zhao et al. (33)	2019	*in-vivo*	48	1,786 cross-sections: 729 non-calcified plaques, 511 calcified plaques, and 546 mixed plaques	N/A
Matsumoto et al. (30)	2019	*in-vivo*	77	118 plaques without extensive calcifications	4 plaques excluded due to extensive echo attenuation on IVUS
Wang et al. (31)	2018	*In-vitro*	N/A	17	N/A
Puchner et al. (29)	2015	*ex-vivo*	3	26 and 67 cross-sections of lipid-core and calcified plaques	Lipid-core plaque was included and defined as any fibroatheroma with a lipid core >60° in circumferential extent, with a core width of >200 μm and a cap thickness of <450 μm
Sun et al. (61)	2018	*In-vitro*	3	3	N/A
Kang et al. (51)	2015	*In-vivo*	42	252 segments of 126 arteries in total, 45 lesions with stenosis ≥25%	N/A
Zreik et al. (53)	2019	*In-vivo*	163	1,259 segments of 534 arteries, 37 non-calcified, 161 mixed and 317 calcified plaques	N/A

In many large-scale studies, the inclusion (or exclusion) criteria and information of subjects are provided in details. To investigate the effects of plaque properties (severity, volume, length, etc.) on the occurrence of myocardial ischemia, a clinical study included 484 coronary arteries extracted from 254 participants. The plaques were categorized according to their severity: 0, 1–29, 30–50, 51–70, 71–90, 91–99, or 100% (56). For machine learning algorithms, it is significant to generate a large-scale dataset for training and validation. Kang et al. investigated the differentiation between obstructive and non-obstructive plaques using machine learning, where CTA data were collected from 42 patients in which 45 stenotic coronary lesions with ≥25% luminal stenosis were extracted from 21 patients (51). Zreik et al. used deep learning to classify coronary plaques (no plaque, non-calcified, mixed, and calcified), where CCTA data of 98 and 65 patients were used for the training and validation of algorithm, respectively. In total, 1,259 arterial segments were extracted from 534 arteries (53). In large-scale studies, the diversity and individual difference in plaque geometry could be comprehensively investigated. However, the data collection for large-scale studies could be time-consuming and expensive.

In the studies on 3D plaque extraction, similar as in 2D studies, the numbers of arterial segments and plaques are generally more than the number of subjects. In some studies, the analysis of plaques is based on the cross-sections, where the number of cross-sections is much higher than that of subjects (29, 32, 33). In a recent study, 1,786 cross-sections were extracted from a CTA dataset of 48 patients to generate enough data for the 10-fold cross-validation of the proposed algorithm (33). The extraction of cross-sections could enlarge the dataset for analysis. Nevertheless, only 2D geometry is represented on cross-sections. Plaque-based analysis is needed to comprehensively evaluate the accuracy of 3D plaque extraction.

#### Classification of Coronary Plaques

The 3D structure of calcified, fibrotic (48), and lipid (49) plaques could be reconstructed from CT images. There are 2 studies which exclusively included non-calcified plaques (64, 72). Two studies were focused on the manual extraction of calcified plaques (61, 62). In these studies, the calcified or non-calcified plaques were not further classified. In the majority of studies on 3D plaque extraction (13 out of 17), both calcified and non-calcified coronary plaques were included (28–33, 47, 51–56). In these studies, we observed diverse standards in the classification of non-calcified plaques, as shown in Table 2. The plaques could be classified according to the main component as soft lipid-rich plaques, mixed plaques, and calcified plaques (55), or non-calcified plaques, low-density non-calcified plaques, and calcified plaques (56). The volume of extracted component heavily depends on the threshold of attenuation value applied in the study (30).

#### Attenuation Values of Different Plaques

As shown in Table 3, in 3D plaque extraction, 130 HU was used as the lower threshold of calcified plaques (28) similar as in 2D studies, while higher attenuation values such as 400 HU (47) and 500 HU (30) were also observed. For non-calcified plaques, the 3D reconstruction studies provided more details on the separation between lipid-rich and fibrous plaques. Matsumoto et al. used different thresholds attenuation (30 and 45 HU) to extract low-density non-calcified plaques (30), and concluded that the upper threshold of 45 HU improved the accuracy of lipid-rich plaque assessment from CTA.

#### Methods of Plaque Extraction and Reconstruction

As in 2D studies, the studies on 3D coronary plaque extraction could be classified as automatic and semi-automatic ones. Manual interactions are common in semi-automatic methods. Additionally, in 3D coronary plaque extraction, there is manual co-registration of IVUS and CTA images (30, 62) which has been applied in detecting vulnerable plaques (76). The details are listed in Table 4.

The boundaries of interested areas can be manually set for 3D analysis. For example, in Gaur et al.'s study, plaque components were quantified within the manually designated area using adaptive algorithms (56).

Manual adjustment of the segmentation and extraction results is common in 3D semi-automatic plaque extraction (29). In Puchner et al.'s study, the vessel wall boundaries obtained by the automated software were reviewed and manually adjusted by an experienced (>5 years of experience in the field of cardiovascular imaging) cardiovascular radiologist. Similarly, in Wang et al.'s study (31), the software automatically traced the plaque boundaries and determined the luminal area, then manual adjustment of the vessel center line and boundaries was performed. In You et al.'s study (28), the plaques were automatically color-coded and manually adjusted. The volume of each plaque component was then automatically measured. In Sun et al.'s study (61), manual editing and image filtering were applied to remove the unwanted structures and smooth the surface of coronary artery lumen.

Manual segmentation results have been widely used in training and validating 3D plaque extraction algorithms. In Sakellarios et al.'s study (52), the initial parameters of their classification mode were set by the median value of the attenuation value of the artery. Manual Expectation-Maximization algorithm based adaption was applied in order to best fit the model to artery's attenuation histogram. In Kigka et al.'s study (47), the result derived by the proposed algorithm was compared with the expert's manual annotation of artery and calcified plaques.

In some studies, multiple manual interactions were used in the semi-automatic 3D reconstruction of coronary plaques. In Wei et al.'s study, after the manual segmentation of arteries, the locations of plaques were manually marked for the training and validation of the algorithm (63). In Athanasiou et al.'s study, the CT and IVUS images were manually co-registered, with the results of manual plaque extraction as the reference for algorithm training (62). In Matsumoto et al.'s study (30), the vessel (external elastic membrane) and lumen contours on IVUS images were manually delineated every 1 mm to calculate plaque volume. Plaque co-registration between CTA and IVUS was performed manually by another investigator, who was not involved in the processing of CTA images. The proximal and distal reference limits of the plaque were matched to IVUS using anatomical landmarks, such as the distance from the aorto-coronary ostium, target lesions, side branches, or calcifications.

In automatic 3D plaque extraction, manual segmentation of the region of interest and marking of the proximal and distal endpoints of plaques (32, 33) have been applied, while the manual segmentation results for algorithm training and validation are more commonly observed (33, 51, 53, 55, 64). Especially, the Rotterdam database provided experts' manual annotations of plaques as the ground truth. The motive behind using Rotterdam data is the availability of the manual ground truth in terms of expert annotations i.e., segment- wise status (normal/abnormal) and the precise position of non-calcified plaque for the abnormal coronary segments. Therefore, it provides a reliable source of reference data for the development of new plaque extraction algorithms (33, 64).

Manual extraction is important for automatic methods based on machine learning. In Zreik et al.'s study (53), plaque type and anatomical significance of the stenosis were manually annotated by an expert using custom-built software following the guidelines of the Society of Cardiovascular Computed Tomography (SCCT) for reporting CAD. Kang et al. used the consensus of three experts' visual assessment as the reference datasets for the 10-fold cross-validation of a structured learning technique to detect all coronary arterial lesions with stenosis ≥25% (51).

Park et al. developed an automatic 3D plaque quantification algorithm and compared the results derived by automatic and semi-automatic methods (54). The results of the automatic algorithm were also compared with the IVUS results for validation. In the semi-automatic method, the boundaries of inner lumen and outer vessel wall were manually edited when needed. Both experts and non-experts participated in the manual segmentation of plaques. While both expert and non-expert groups used automatic centerline extraction, the experts edited the inner lumen and the outer vessel wall contours manually, whereas, the non-expert readers used the longitudinal contours for manual manipulation with minimal cross-sectional editing. Lastly, the analysis was performed on the same segments using the fully automatic contour detection algorithm without manual editing. The automatic and semi-automatic methods derived comparable results in plaque quantification analysis.

#### Technical Innovations

For data processing and analysis, most of the 3D reconstruction used specific algorithms whose names are disclosed while 2 studies used the algorithms embedded in the software (28, 54).

Park et al. used QAngio CT Research Edition (v2.02; Medis medical imaging systems bv, Leiden, The Netherlands) for the semi-automatic and automatic quantitative CT analysis (54). The 3D reconstruction started with an automatic centerline extraction. Based on these centerlines, straightened multiplanar reformatted (MPR) volumes were reconstructed for the segmentation and quantification. Longitudinal inner lumen and outer vessel wall contours were detected by an automatic algorithm and were segmented automatically in the transversal images. The extracted geometry was then reviewed by experts, and manually edited if necessary (54).

You et al. combined different novel algorithms embedded in software in 3D plaque reconstruction (28). The 15-segment coronary arterial model proposed by AHA was adopted to select the arteries with diameter >2.0 mm for further analysis, with blurred segments excluded. Maximum intensity projection, volume rendering, multiplanar reformation, and curved multiplanar reformation results were routinely constructed using the algorithms embedded on a commercial workstation (EBW, Philips Medical Systems). If an abnormal segment was identified, that coronary artery was evaluated on an Aquarius workstation (TeraRecon, San Mateo, CA) where non-calcified plaques were divided into lipid-rich (0–49 HU) and fibrous (50–129 HU) plaques. The lesions on the baseline and follow-up images were matched using adjacent anatomical landmarks. The CAC Agatston calcium scores were calculated using semi-automated software (EBW; Philips Medical Systems, Best, The Netherlands), which identified the areas of at least 0.5 mm^2^ and a density ≥130 HU on CT images as calcification (28). The authors concluded that the application of different embedded algorithms could get the analysis results in a relatively short period for clinical use.

FBP is the current standard CT image reconstruction technique (30), therefore, it is widely used as the reference for the validation of 3D (29, 32) and 2D (39) plaque reconstruction algorithms. Nevertheless, FBP is sensitive to the large variations in attenuation value between pixels caused by noises, with the quality of reconstructed plaques affected (77).

Compared with FBP, IR algorithms are more robust in the existence of noises. Hybrid IR could reduce the noise or artifacts in CT images (32) and improve the quality of low-dose chest CT images compared with FBP (77). Especially, Model-Based IR (MBIR) uses optic parameters of the CT scanner to improve the imaging quality, and has been applied in the reconstruction of medical images in low radiation dose (31, 78). As in 2D studies, the IR algorithms consist the majority of new algorithms in 3D reconstruction of coronary plaques.

Takahashi et al. compared ASIR, MBIR, and FBP algorithms in extracting calcified and non-calcified plaques from the CCTA images of 352 patients (29). They found that image noise, Agatston score, and calcium volume decreased significantly with ASIR compared to FBP (each *p* < 0.001) (79). MBIR had higher accuracy in detecting lipid-core plaques on CCTA images compared with FBP and ASIR (*p* = 0.01, in 173 cases).

In Károlyi et al.'s study (32), compared with FBP and HIR, IMR derived the highest CNR (*p* < 0.01), and the lowest overall plaque volumes as well as calcified (>130 HU) volumes (*p* < 0.05 for all). For non-calcified plaques, compared with FBP and HIR, IMR derived lower high-attenuation non-calcified volumes (90–129 HU) (*p* < 0.05 for both), but similar intermediate- (30–89 HU) and low-attenuation (<30 HU) non-calcified volumes (*p* > 0.05 for all).

Different 3D reconstruction algorithms could lead to different hemodynamic parameter estimations. The computational fluid dynamics (CFD) simulation on 3D-reconstructed coronary artery models showed that the FFR values derived from the 3D coronary artery models reconstructed by FBP and iterative reconstruction in image space (IRIS) are different but linearly related [*r* = 0.74, 0.76, and 0.70 in left main coronary artery (LMCA), LAD, and RCA] (80).

In 3D plaque extraction, some automatic methods have been proposed based on machine learning or deep learning algorithms including convolutional neural network (CNN), Support Vector Machine (SVM), and Gaussian Mixture Model (GMM).

CNN is the commonest architecture in cardiovascular image analysis (81). Zreik et al. investigated the automatic detection and classification of plaques using a multi-task recurrent convolutional neural network (RCNN) (53). Centerlines of the coronary arteries were extracted from CCTA images of 163 patients to reconstruct MPR images. The type (no plaque, non-calcified, mixed, calcified) and anatomical significance (no stenosis, non-significant, i.e., <50% luminal narrowing, and significant, i.e., ≥50% luminal narrowing) of plaques in the coronary arteries were manually annotated in the MPR images as the reference. To perform an automatic analysis, a multi-task RCNN was applied on the MPR images of coronary arteries using cubes of 25 ×25 ×25 voxels. The network was trained and tested using the CCTA images of 98 and 65 patients, respectively. In detecting the plaque type and anatomic significance, the method achieved the accuracy of 0.77 and 0.80, respectively. Authors concluded that CNN algorithm could be applied in the automatic detection and classification of coronary artery plaques, which could benefit the automated triage of CAD patients (53).

SVMs are supervised machine learning techniques. An SVM achieves the classification by constructing a multidimensional hyperplane that optimally discriminates between two classes, by maximizing the margin between two data clusters. Support Vector Machine has been widely used in the reconstruction of different organs from CT images (82). Zhao et al. proposed an automatic multi-class coronary atherosclerosis plaque detection and classification framework based on SVM. Firstly, the transverse cross-sections were retrieved along centerlines in CCTA images, with the region of interest extracted by coarse segmentation. Secondly, a random radius symmetry (RRS) feature vector was extracted, which incorporated multiple descriptions into a random strategy and greatly augmented the training data. Finally, the RRS feature vector was fed into the multi-class coronary plaque classifier. The proposed SVM-based algorithm outperformed intensity feature vector and the random forest classifier on the Rotterdam Coronary Datasets which includes 729 non-calcified plaques, 511 calcified plaques, and 546 mixed plaques (average precision: 92.6%) (33).

Kang et al. developed a robust automated algorithm of plaque detection based on SVM (51). All coronary arterial lesions with stenosis ≥25% were detected by a structured learning technique. The plaque detection algorithm consists of two stages: (1) two independent base decisions indicating the existence of lesions in each arterial segment based on SVM and formula-based analytic method and (2) the final decision made by combining the base decisions. The SVM algorithm extracted the geometric and shape features from small volume patches of arterial lesions. On 42 CTA patient datasets where 21 datasets had 45 lesions with stenosis ≥25%, the proposed method achieved high sensitivity (93%), specificity (95%), and accuracy (94%), with consensus reading of lesions with stenosis ≥25% by three expert readers as the reference. Authors concluded that their SVM-based algorithm was promising for automated detection of obstructive and non-obstructive lesions from CTA images (51).

A GMM is a probabilistic model based on a Gaussian distribution for expressing the presence of sub-populations/sub-classes within an overall population/class without requiring the identification of the sub-class of interest (observational data). The GMM specifies the features of the clusters which indicate different tissues, and estimates which features are likely to differ between clusters (83).

Sakellarios et al. proposed a 3D reconstruction method based on the Radial Intensity Projection (RIP) (52). At each equally distant (2 mm) point on the centerline, a radial image was produced perpendicular to the centerline using the B-spline derivatives extracted at the specific point. The centerline was modified using an iterative radial image correction to avoid surface intersections in highly curved segments. Lumen, calcified plaque, and non-calcified plaque were modeled as a 3-component GMM. The initial parameters were set by the median value of the HU intensity of the artery. Manual or automated Expectation-Maximization algorithm based adaption was applied in order to best fit the model to artery's HU histogram. Using the GMM model, each pixel was classified to one of the five classes: (i) inner wall, (ii) outer wall, (iii) calcified plaque, (iv) non-calcified plaque, and (v) background, based on the class/component with the maximum posterior probability. The algorithms were integrated into a tool for semi-automatic extraction of coronary and carotid arteries (52). Similarly, in Athanasiou et al.'s work, the Perpendicular Radial Image (RPI) was generated along the centerline for the detection of lumen wall and potential plaque lesion borders (62). Based on the attenuation values, the PRI image was classified into lumen, non-calcified plaque, calcified plaque, and background pixels using a 4-component GMM. The parameters of the GMM were fitted to each CT dataset, based on a set of regions (from each dataset) manually annotated by an expert to lumen, non-calcified plaque, calcified plaque, and background.

Jawaid et al. proposed a hybrid energy model to extract the coronary artery tree. A tubular model and an elliptical model were used to present the geometry of arterial segment and cross-sections, respectively. The boundary of stenosed segment was reconstructed from adjacent normal segments. The reconstructed non-calcified plaques were compared with the manually extracted lumen deformations. This automated plaque segmentation method achieved the accuracy equivalent to human experts, but a bulk of data is needed for adequate training of the CNN (64).

The level-set model could simplify the numerical computations of curves and surfaces in the 3D reconstruction of plaques. Kigka et al. developed a semi-automated method using level sets to extract calcified and non-calcified plaques as well as arterials walls (47). The results were in accordance with the manual annotation by experts and the results reconstructed from IVUS images.

Motion artifacts could cause the deformation of the reconstructed 3D arterial geometry. To eliminate the artifact-defective segmentation, Ghanem et al. proposed a robust framework for the 3D reconstruction of coronary arteries. Firstly, the initial contour of lumen inner wall was derived using Hessian analysis and region growing. Secondly, the initial contour of arterial outer wall was derived using mathematical morphology. Finally, the lumen and vessel wall were segmented using level sets. Based on the extracted geometry, the 3D meshes of lumen and vessel wall were generated using marching cube methods. Curved multi-planar reformation was used to modify the geometry (55).

#### Geometric Parameters in Measurement

As summarized in Table 6, the 1D and 2D geometric parameters used in 2D plaque extraction could also be measured in 3D plaque extraction. The severity of stenosis and plaque burden could therefore be calculated as in 2D reconstruction. The severity of stenosis could be estimated by the ratio of lumen diameters at the stenotic center (Ds) and normal segment (Dn): severity = 1-Ds/Dn. This parameter reflects the thickening of arterial wall due to the accumulation of adipose tissue, and is directly related to the decrease in myocardial blood flow (53). Kang et al. proposed a new parameter to evaluate the shape of an arterial cross-section from its area and perimeter: *circularity* = 4π·*area/perimeter*^2^ (51). Additionally, the maximal lumen area stenosis percentage was also used to estimate the severity of stenosis (54). For the lipid-rich non-calcified plaques, the percentage of the lipid core on the arterial cross-sections and cap thickness (μm) were measured (29) to evaluate the extent of lipid core development.

**Table 6:** Geometric parameters in CT-based coronary plaque evaluation.

**Study**	**Geometric parameters**	
	**2D**	**3D**
Gaur et al. (56)	Numbers of obstructive lesions (stenosis > 50%), plaque length (mm).	Volume of non-calcified plaques; low-density non-calcified plaques, calcified plaques, and all plaques (mm^3^), aggregate plaque volume (%).
Jawaid et al. (64)	Arterial wall thickness, lumen area.	N/A
Sakellarios et al. (52)	Artery outer border, area of plaques on cross-sections.	Centreline, volume.
You et al. (28)	N/A	Volume of calcified, fibrous and lipid-rich plaques (mm^3^)
Ghanem et al. (55)	Vessel wall thickness (mm), plaque length (mm), luminal stenosis (%),	Volume of calcified, mixed, and soft lipid-rich plaques (mm^3^)
Wei et al. (63), as summarized in the review by Jawaid et al. (72)	N/A Stenosis volume in %	Centreline and length of vessels
Athanasiou et al. (62)	N/A	Volume (mm^3^), surface area (mm^2^), maximum length (mm), and inner angle (degree) of plaques. Overlapping volume between different objects (lumen, wall and plaque).
Park et al. (54)	Minimal lumen area (mm^2^), maximal lumen diameter stenosis percentage (%), maximal lumen area stenosis percentage (%), mean plaque burden (%).	Volume of lumen, vessel, and plaques (mm^3^)
Kigka et al. (47)	Degree of stenosis (%). Minimal lumen diameter (mm). Minimal lumen area (mm^2^). Plaque burden (%).	N/A
Károlyi et al. (32)	Lesion length (mm)	Plaque volume (mm^3^), lumen volume (mm^3^), vessel volume (mm^3^).
Zhao et al. (33)	Estimated radius of plaque area segmented from cross-section (mm).	N/A
Puchner et al. (29)	Area of calcified and lipid-care plaques on cross-sections (mm^2^), circumference, width, and cap thickness of lipid core (μm), plaque burden (%)	N/A
Wang et al. (31)	Lumen area (mm^2^)	volume of plaque components (mL) and their relative values (%)
Matsumoto et al. (30)	Plaque thickness (mm), plaque area (mm^2^) on cross-section, Plaque burden (%).	Plaque composition volume (mm^3^) and the ratio in total plaque volume
Sun et al. (61)	Degree of lumen stenosis (%).	N/A
Kang et al. (51)	Degree of lumen stenosis (%). Circularity of cross-section.	Location of stenosis (mm) from ostium.
Zreik et al. (53)	Degree of lumen stenosis (%).	N/A

**Figure 1:**
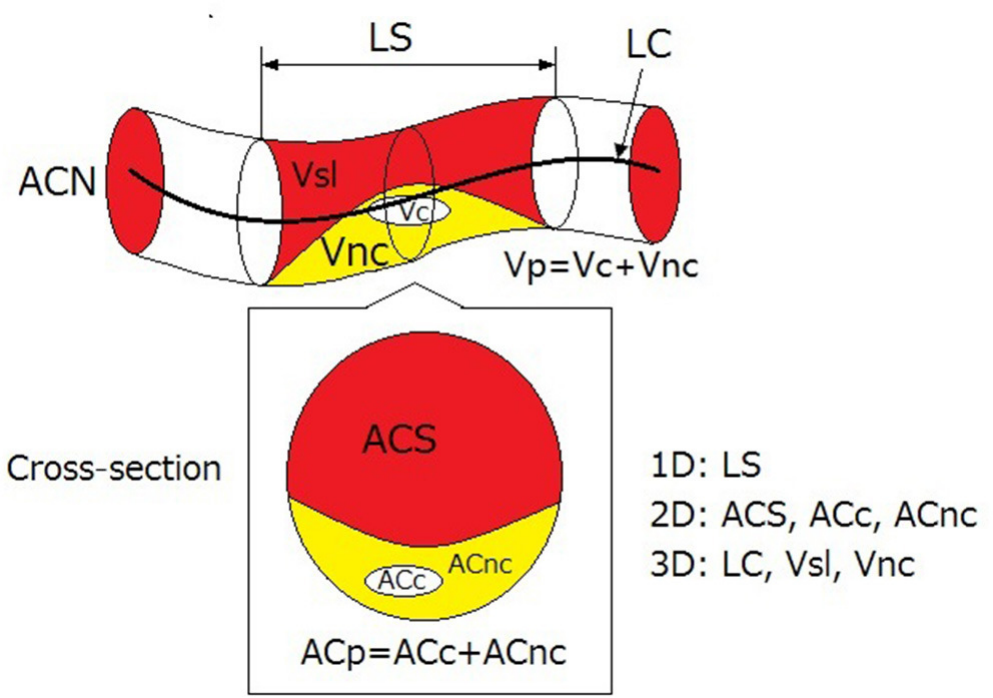
1D, 2D, and 3D geometric parameters used in 3D plaque reconstruction. LS, length of stenosis; LC, length of centreline of arterial segment; Vsl, volume of lumen in stenosed segment; Vnc, volume of non-calcified components; Vc, volume of calcification; Vp, volume of plaque; ACN, area of lumen cross-section in normal segment; ACS, area of lumen cross-section in stenosed segment; ACc, cross-section area of calcification; ACnc, cross-section area of non-calcified (lipid or fibrotic) components; ACp, cross-section area of plaque. The red, yellow, and white areas illustrate the stenosed lumen, the non-calcified components, and the calcification.

Besides aforementioned 1D and 2D parameters, some 3D geometric parameters could be measured in 3D plaque extraction, including the volume of plaque and different components, the length of arterial segment centerline, surface area, and the angle between the vector of centerline and plaque surface (62). Figure 1 illustrates the geometric parameters commonly used in 3D plaque extraction.

#### Intra- and Inter-Observer Repeatability

In the 16 original studies on 3D plaque extraction, 4 included the intra-observer repeatability (32, 33, 54, 55), and 8 included the inter-observer repeatability (28, 30, 32, 51, 53–56).

The repeated measurements were performed by experts including experienced radiologists or technicians (32). Most of these studies included 2 experts on coronary imaging. Exceptionally, a study included three expert readers of CT images to make a consensus reading as the reference which was compared with the reading made by a blinded reader (51). It was suggested that intra- and inter-observer repeatability is important for algorithm validation and has been widely used in recent studies on 3D plaque extraction (72).

#### Reference of Accuracy

As in 2D studies, IVUS and ICA were used as the reference for the evaluation of 3D plaque extraction methods (39, 64). In addition, different types of scans such as MTCT or biplane X-ray angiography have also been used as the reference of accuracy (47). One study reported using 3D remodeling, which is a relatively new method for the coronary plaque assessment (64).

## Discussion

### Summary: Merits and Limitations of Current Methods

In this review, we focus on the summarization of the merits and limitations of recent studies in three aspects (data, method, and evaluation), not the detailed analysis of algorithmic innovations. The methods and algorithms in these studies are highly diverse with different theoretical bases. Therefore, we introduced some innovations in these methods and algorithms but did not list the details.

#### Data

In both 2D and 3D studies, *in vivo* data were commonly used. *In vivo* data reflect the patient-specific anatomical structures, therefore are indispensable for the full validation of plaque extraction algorithms. However, for *in vivo* data, motion artifacts caused by cardiac movements could affect the quality of CT images. In comparison, motion artifacts are excluded from *ex vivo* data. Therefore, *ex vivo* data could be used to evaluate the accuracy of algorithms in reflecting anatomic details. Regarding *in vitro* and phantom data, geometric parameters could be directly measured on the models; therefore the accuracy of plaque extraction algorithms could be evaluated quantitatively. For phantom data, geometric parameters such as the radius of arterial segment, the thickness of plaque, as well as the material components, are all adjustable for the evaluation in different levels. Additionally, biomechanical or hemodynamic experiments could be performed on the *in vitro* models and phantoms to evaluate the plaque extraction algorithms in different pathophysiological conditions.

Most of the reviewed studies used 1-2 types of data. More types of data can be included for the comprehensive evaluation of new algorithms in geometrical details. The majority of studies included <100 human subjects. Considering the individual difference in the anatomy of coronary arteries, multi-center large-scale studies are necessary to validate the proposed algorithms for clinical applications.

#### 2D and 3D Algorithms

The majority of 2D reconstruction methods are based on FBP and IR. The machine learning algorithms are widely used in the 3D reconstruction of coronary plaques (33, 51–53, 62).

IR algorithms could reduce noises and radiation dose in CT scanning, and improve the quality of CT images of obese patients, coronary atherosclerotic plaques, coronary stents, and myocardial perfusion (84). Therefore, IR algorithms have been widely embedded in the software. Despite the benefits in dose reduction, it is still unclear exactly which kV and mAs for a given body habitus is optimal with each IR algorithm (85). Furthermore, IR could influence many factors that are important for the clinical risk stratification of CAD, including coronary calcification, plaque burden and composition, as well as stenosis severity (85). There is a lack of comprehensive evaluation of different IR algorithms regarding diagnostic accuracy and patient management. In most of the studies included in this review, the results of IR algorithms were compared with the results of standard FBP method only (see sections Technical Innovations). Additionally, there is a lack of quantitative evaluation of IR algorithms based on *ex vivo* or phantom data.

Tsompou et al. compared the 3D reconstruction methods based on different cardiovascular images (86). It was found that, with de-blooming algorithms, the geometric parameters (normal and stenosed lumen diameters, severity and length of plaque) and wall shear stress calculated from the 3D models reconstructed from CCTA images were not significantly different from the results derived from quantitative coronary analysis and IVUS (86). Therefore, the accurate estimation of 3D plaque geometry could be achieved by using CT images. However, the majority of 3D plaque reconstruction algorithms are based on attenuation value or diameter estimation (Table 3). There is a lack of investigation on the 3D geometric characteristics of coronary plaques.

Machine learning has been widely used in the analysis of cardiac images and signals and has been proven to be effective in predicting heart failure and other clinical events (87). Especially, fully automated machine learning algorithms may facilitate the processing of large-scale image datasets. For clinical application, images from picture archiving and communication systems can be segmented out and fed into other machine learning layers in order to establish a diagnostic and a prognostic course (87). For example, the risk of plaque rupture is associated with stress concentration, which depends on the mechanical properties and the geometry of the reconstructed plaques (88). Thus, machine learning could assist the clinical professionals to estimate the vulnerability of the atheroma plaque (88). In comparison, traditional 3D finite element analysis of plaque rupture requires huge computational resources, therefore is not suitable for clinical use. The machine learning methods have been applied in the classification of plaque type (33, 53) and anatomic significance (51, 53, 64), whereas, there is a lack of clinical validation and application. Additionally, machine learning can be used in the estimation of hemodynamic parameters of coronary arteries such as FFR from CTA images (89). The application of machine learning in the CT-based coronary plaque assessment deserves further investigation under multidisciplinary collaboration.

#### Automatic Algorithms and Manual Interactions

As shown in Table 4, setting the boundaries and adjusting the results are the commonest manual interactions in semi-automatic plaque extraction methods (72). However, manual adjustment is time-consuming and dependent on operator skills (24). There is a high need to develop automated methods that can achieve the reliable extraction of coronary plaques.

Advanced algorithms based on AI (machine learning, deep learning, etc.) provide an important approach toward the automation of coronary plaque extraction. For example, Wolterink et al. have successfully developed an automatic method to identify the calcified voxels using paired convolutional neural networks (90). Furthermore, based on the big data and new technologies such as radiomics, more information could be extracted in parallel with the reconstruction of plaque geometry, achieving the preliminary diagnosis and automatic screening of CAD patients based on clinico-radiological information (91).

In both semi-automatic and automatic methods of plaque extraction, the manual extraction results have been widely used for training and validating the algorithms. In 2013, Kirisli et al. compared 11 automatic and semi-automatic algorithms of coronary plaque extraction on a dataset of 48 symptomatic CAD patients (92). The authors quantitatively evaluated the accuracy of these algorithms. They concluded that current stenosis detection/quantification algorithms are not sufficiently reliable to be used stand-alone in clinical practice, but that some could be used for triage or as a second-reader. They also suggested that automatic lumen segmentation could achieve the precision comparable to experts' manual segmentation. Thus, the manual extraction results with high accuracy still play a key role in algorithm evaluation. The standardized datasets such as Rotterdam dataset (33, 64) provided an appropriate choice. Nevertheless, due to the limitations in data sharing, very few datasets are currently available.

IVUS images have high resolution, which makes them adequate for clinical diagnosis and algorithm validation. Additionally, to improve the accuracy of plaque extraction, IVUS images could be used as a virtual reality tool to explore and understand the outer and inner structure of coronary arteries (76). Compared with manually extracted results, IVUS images could serve as a more reliable reference for the validation of plaque extraction algorithms. However, the validation requires the co-registration of CT and IVUS images, which is still often performed manually.

#### Geometric Parameters

The geometry of coronary arteries and plaques could influence the development of atherosclerosis and the occurrence of cardiovascular events (93). In the majority of 2D studies, the geometric parameters are measured from the cross-sections or other 2D images. In 3D studies, 1D, 2D, and 3D parameters are all included. It has been proven that the geometric parameters (cross-section area, area severity, etc.) of coronary plaques are reproducible with high intra- and inter-observer agreement (94). The severity of plaques has been estimated by the ratios in diameter (53) and area (39, 66). Some secondary parameters could be derived from the 3D geometry of coronary plaques. For example, radius gradient of the plaque, which reflects longitudinal lesion asymmetry, has been proven to be associated with the location of plaque rupture and consequent clinical events (95). The curvature and tortuosity of coronary arteries might be related to the development of atherosclerosis and plaque size (96, 97). These secondary parameters and their clinical indication need further investigation.

#### Extraction and Classification of Different Plaques

As to the extraction of calcified plaques, de-blooming algorithms (17, 86) have been proposed and proven to be effective in reducing blooming artifacts (86). However, it was found that the calcium density, which is directly related with blooming artifacts, has little effect on the accuracy of CTA (98). Therefore, other details such as the dose, individual difference, and the de-blooming of co-existing plaques and stents (99), need further investigation to improve the accuracy and reliability of calcified plaque extraction.

**Figure 2:**
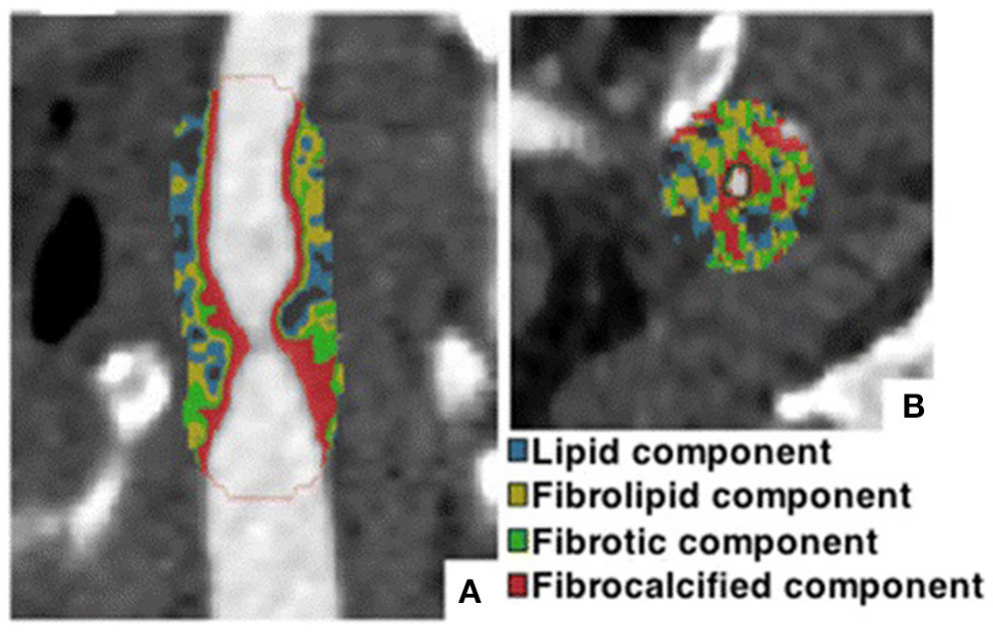
Extraction of different components of a vulnerable plaque in carotid artery using semi-automatic method based on different HU values. **(A)** Sagittal section of carotid artery. **(B)** Transverse section. Adapted from Diab et al. (100) ^©^ 2019 by the authors. CC BY 4.0.

For non-calcified plaques, we observed different classification standards (Table 2) and attenuation thresholds (Table 3). The most detailed classification included lipid-rich, fibrous, and calcified plaques. In a parallel study on plaque extraction in carotid arteries on CTA images, the components of non-calcified plaques have been classified as lipid, fibrofatty, fibrotic, and fibro-calcified (100) (Figure 2). In another study, carotid plaques are classified as intimal lipid accumulation, lipid-rich necrotic cores, calcification, fibrosis, and calcification (101). Lipid-rich necrotic core is a major characteristic of high-risk vulnerable plaques, which is an important reference for clinical diagnosis and intervention. It has been known that lipid-rich lesions can be separated from more fibrous ones on CT images, which could be used to estimate the risk of plaque rupture (102). In 2013, Obaid et al. evaluated the accuracy of a 3D plaque extraction method in estimating different components of coronary plaques on CT images (103). The accuracies of CT and VH-IVUS were comparable in detecting calcified plaque (83 vs. 92%), necrotic core (80 vs. 65%), and fibroatheroma (80 vs. 79%), with *ex vivo* histology as the reference. A plaque containing large amounts of lipid may be classified by VH-IVUS as fibro-fatty tissue but have low attenuation, and be classified as the necrotic core on CT images. Accurate and quantitative estimation of different components of non-calcified plaques on CT images is still challenging due to the limited temporal, spatial, and contrast resolutions of current scanners (102).

#### Future Directions

In future studies, the application of machine learning and automatic methods (extraction of the centerline of coronary arteries, segmentation, quantification of calcification and other components, etc.) could improve the efficiency of coronary plaque extraction from CT images. More geometric parameters could be derived from the 3D geometry of extracted plaques. The accuracy of plaque extraction could be improved in the following aspects: the inclusion of more data types, the comprehensive evaluation of IR algorithms on *ex vivo* and *in vitro* data, the multi-center large-scale studies, more standardized datasets, the investigation on the geometric properties of coronary plaques, further investigation and standardization of de-blooming algorithms, and more detailed classification of non-calcified plaques.

## Author Contributions

HL proposed the structure of the review. AW searched for the literature. HL and AW analyzed the literature and drafted the manuscript. DZ supervised the project that led to production of the results. All contributed to the discussion and revision of the manuscript and concur with the current submitted version.

## Conflict of Interest

The authors declare that the research was conducted in the absence of any commercial or financial relationships that could be construed as a potential conflict of interest.
